# Defining Potential Neurovascular Risk Zones in Superficial Plantar-Medial Release: An Anatomical Study

**DOI:** 10.3390/jpm16060302

**Published:** 2026-06-03

**Authors:** Elisabeth M. Mandler, Zehra Düzgün, Johannes M. Mittendorfer, Jakob R. Altmann, Lena Hirtler

**Affiliations:** Center for Anatomy and Cell Biology, Medical University of Vienna, 1090 Vienna, Austria; elisabeth.mandler@meduniwien.ac.at (E.M.M.); zehra.duezguen@meduniwien.ac.at (Z.D.); johannes.mittendorfer@meduniwien.ac.at (J.M.M.);

**Keywords:** superficial plantar medial release, abductor hallucis, tibial nerve, posterior tibial artery, plantar fascia, plantar aponeurosis, plantar fasciitis, pes cavus, cavus foot, plantar nerve, plantar artery

## Abstract

**Background:** The superficial plantar-medial release (S-PMR) refers to a group of surgical procedures involving the release of the plantar aponeurosis and adjacent medial plantar soft tissues that are used in selected cases of plantar fasciitis and cavovarus foot deformity. The procedure aims to address pain and contracture of the plantar aponeurosis and intrinsic foot muscles, which may contribute to pathological foot alignment and gait instability. Due to the close proximity of highly variable neurovascular structures in the plantar region, precise anatomical knowledge and a patient-specific, personalized approach are essential to reduce the risk of iatrogenic injury during surgery. This study defined procedure-specific anatomical “low-risk” and “high-risk” zones. **Methods:** From the initial forty-two included feet, one specimen was excluded due to insufficient tissue quality, leaving forty-one specimens for analysis. The plantar aponeurosis, origins of the abductor hallucis muscle and regional neurovascular structures were analyzed. Distances between key landmarks were measured. **Results:** Abductor hallucis origins I and IV were present in all specimens, while origins II and III showed variable presence. Subdivision of muscle origin I was observed and was associated with the course of the medial calcaneal nerve. The medial calcaneal nerve demonstrated the closest proximity to origin I (3.2 mm) whereas both the medial and lateral plantar nerves showed close proximity to origin II (3 mm and 5.3 mm). **Conclusion:** Significant interindividual variability exists in the plantar region, highlighting the need for a personalized, anatomy-based approach for patients considered for surgical intervention. Anatomical “high-risk” zones were identified between origin I and the medial calcaneal nerve and near origin II by the bifurcation of the tibial nerve and posterior tibial artery. Anatomical “low-risk” zones were defined as dorsal regions at the calcaneus between origin I and the tibial neurovascular bundle, as well as medial areas near the malleolus.

## 1. Introduction

The superficial plantar-medial release (S-PMR) is a surgical procedure involving targeted soft tissue release of the plantar aspect of the foot to alleviate pain or to treat gait instability associated with specific pathologies, most notably plantar fasciitis and cavus foot deformities [1,2]. Plantar fasciitis is a degenerative condition of the plantar aponeurosis (or plantar fascia) and represents the most common cause for heel pain [3]. Standard management is primarily conservative, typically extending over 9–12 months, with surgical intervention reserved for patients who do not respond to prior treatment [4,5]. In contrast, cavovarus foot deformity constitutes a complex three-dimensional structural abnormality characterized by an elevated and concave medial longitudinal arch, inversion of the hindfoot and plantar flexion of the first metatarsal [6,7]. This condition falls within the broader spectrum of cavus foot deformities and can necessitate surgical correction due to its progressive and multifactorial nature [8].

Depending on the underlying pathology and surgical indication, S-PMR may involve partial release of the plantar aponeurosis, components of the abductor hallucis muscle origin, or a combination of both [9,10,11]. The origins include the medial process of the calcaneal tuberosity, the flexor retinaculum and the plantar aponeurosis [12]. The procedure can be performed using an open or endoscopic approach [13]. Despite its therapeutic benefits, S-PMR carries potential risks, including neurovascular injury or entrapment, delayed healing, soft tissue infection, persistent postoperative pain and reduced plantar heel sensation [14,15].

A thorough understanding of the regional anatomy, including potential anatomical variations, is critical for minimizing surgical complications. Multiple neurovascular structures traverse the surgical field in close proximity to the targeted release zones [4,15]. Of particular importance are the branches of the tibial nerve, including the medial and lateral plantar nerve, as well as the medial and inferior calcaneal nerves. Additionally, the medial and lateral plantar arteries, which typically arise from the posterior tibial artery, are at risk during surgical intervention in this area [9,12]. Iatrogenic injury of these structures may result in loss of plantar sensation, intrinsic muscle weakness, altered gait mechanics, chronic neuropathic pain, and intra- or postoperative bleeding [16,17,18,19]. Despite the clinical relevance of this region, detailed anatomical descriptions of the variability of the abductor hallucis origins and their spatial relationship to adjacent neurovascular structures remain limited.

Therefore, the present study aims to investigate the anatomy of the relevant neurovascular structures in relation to the origins of the abductor hallucis muscle, flexor retinaculum and plantar aponeurosis. Furthermore, it seeks to define surgical “low-risk” and “high-risk” zones for the S-PMR, thereby contributing to improved intraoperative guidance in order to optimize protection of neurovascular structures.

## 2. Materials and Methods

Twenty-one paired fresh-frozen feet from human body donors (n = 42) originating from the Center for Anatomy and Cell Biology, Medical University of Vienna, Austria, were examined. All donors had given informed consent for their bodies to be used post mortem for scientific purposes and teaching. The study was approved by the Ethics Committee of the Medical University of Vienna (EK No.: 1011/2023). Exclusion criteria included evidence of prior surgery, relevant trauma, or insufficient tissue quality. One specimen had to be excluded, which led to a sample size of 41 foot-ankle specimens (17 feet of male and 24 feet of female body donors). The average donor age was 78.5 (54–94) years; 57.1% of the donors were female (n = 12) with an average age of 73 (54–93) years, and 42.9% were male (n = 9) with an average age of 84 (73–94) years.

A longitudinal incision was performed along the medial hindfoot, extending from the calcaneal tuberosity to the posterior neurovascular bundle located posterior to the medial malleolus and proximal to the posterosuperior margin of the flexor retinaculum. The flexor retinaculum was incised vertically to expose the underlying neurovascular structures. Due to inconsistent descriptions of the origin of the abductor hallucis in the literature, the number and spatial arrangement of its muscle origins were documented and categorized prior to muscle detachment. Classification of the muscle origin began at the most distal point of the calcaneus. Origins were defined as macroscopically distinguishable attachment areas separated by visible connective tissue planes or neurovascular structures. A sub-origin was recorded when a discrete separation within the muscular attachment could be identified. Following identification of the muscular structures, the regional neurovascular structures, including the medial and lateral plantar nerves and arteries as well as the medial and inferior calcaneal nerves, were exposed. Distance measurements were obtained between surgically relevant reference points considered potentially vulnerable during S-PMR procedures. Distances were recorded between the closest margins of the respective anatomical structures. All measurements were performed using an analog caliper (accuracy: 0.02 mm). Measurements and dissections were performed by a single investigator following standardized dissection and measurement protocols. Formal intraobserver and interobserver reliability analyses were not performed due to the exploratory anatomical nature of the study. Negative values were assigned when neurovascular branching occurred distal to the superior border of the flexor retinaculum. These measurements were used to assess the spatial relationship between muscular attachments and adjacent neurovascular structures with the aim of identifying regions of relatively close or distant neurovascular proximity relevant to S-PMR procedures. Statistical analyses were conducted using R Studio^®^ software (2025.09). Distributional assumptions were assessed using the Shapiro–Wilk test. Depending on the data, exploratory group comparisons were performed using two-tailed Student’s *t*-tests or Mann–Whitney U tests. Nominal variables were analyzed using Chi-square tests, and correlations were evaluated using Spearman’s rank correlation coefficient. Normally distributed variables are presented as mean ± standard deviation, whereas non-normally distributed variables are presented as median values with full ranges. Statistical significance was set at *p* < 0.05. As this was an exploratory cadaveric anatomical study, no a priori sample size or power calculation was performed. The available number of suitable specimens determined the final sample size. Importantly, as paired specimens from the same donors were included without accounting for intra-donor dependency, the results are of an exploratory nature and should only be interpreted descriptively.

The study was conducted in accordance with the Declaration of Helsinki, and approved by Ethics Committee of the Medical University of Vienna, Austria (protocol code 1011/2023 and approved on 14 February 2023). All donors had given informed consent for their bodies to be used post mortem for scientific purposes and teaching.

## 3. Results

**Figure 1:**
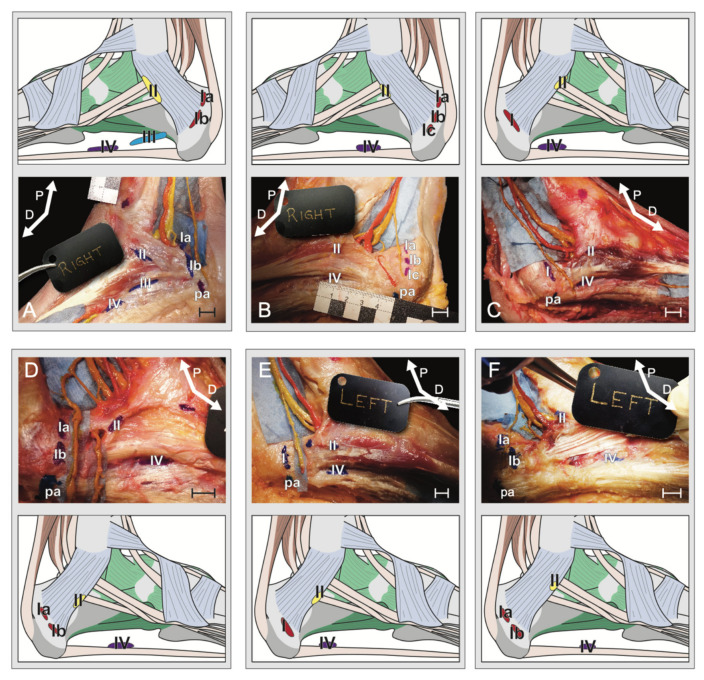
Medial view of six different exemplary foot specimens (**A**–**F**) demonstrating the origins of the abductor hallucis muscle. The images illustrate the variability of origins. For each photograph a schematic illustration is provided to illustrate the abstract location of each origin. Orientation: P = proximal, D = distal. Abbreviations: pa = plantar aponeurosis, I–IV = origins of abductor hallucis I–IV, Ia–Ic = origin I of abductor hallucis with its sub-origins a, b, c.

Foot length, defined as the most dorsal point of the heel to the basal phalanx (insertion of abductor hallucis), measured 20.54 cm (SD = 1.48 cm) in males and 18.67 cm (SD = 1.00 cm) in females. The abductor hallucis muscle demonstrated considerable anatomical variability (see Table 1 and Figure 1A–F).

The first origin (I), arising from the medial aspect of the calcaneal tuberosity, and the fourth origin (IV), attached to the medial aspect of the plantar aponeurosis, were present in all specimens (100%). In contrast, the second muscle origin (II) located at the proximal flexor retinaculum was identified in 31 specimens (76%), whereas the third origin (III) arising from the interfacial septum was observed in 28 cases (68%). Muscle origin diversification only affected origin I (see Table 1) and was observed in 12 cases (29%), resulting in up to three sub-origins (Ia–Ic) (see Figure 1B). Triple subdivision was observed in 5% of specimens (n = 2). Division of origin I can mainly be attributed to the frequent presence of a branch of the medial calcaneal nerve or a branch of the accompanying artery in this region, which was typically very delicate and exhibited considerable variability in its location. Within both female and male specimens, no differences in the frequency distribution of the four muscle origins (I–IV) were observed in this study (females: χ^2^ = 0.95, *p* = 0.8; males: χ^2^ = 3.78, *p* = 0.29).

To identify potential “low-risk” and “high-risk” zones, distance measurements between the main muscle origins and six adjacent neurovascular structures were taken and are summarized in Table 2 and Table 3.

Origin I showed the closest proximity to the medial calcaneal nerve (median = 3.2 mm). Origin II was located near multiple neurovascular structures, with the medial and lateral plantar nerves being closest (3.0 mm and 5.3 mm). In contrast, origin III was mainly associated with medial neurovascular structures, particularly the medial plantar nerve (5.0 mm), whereas origin IV showed closest proximity to the lateral plantar nerve and artery (2.6 mm and 5 mm). Of particular surgical relevance was the relationship between plantar aponeurosis and origin I of the abductor hallucis, as both structures are frequently transected during S-PMR surgery. Across all measurements between origin I and the plantar aponeurosis, the mean distance was 4.6 mm (SD = 3.7 mm).

**Figure 2:**
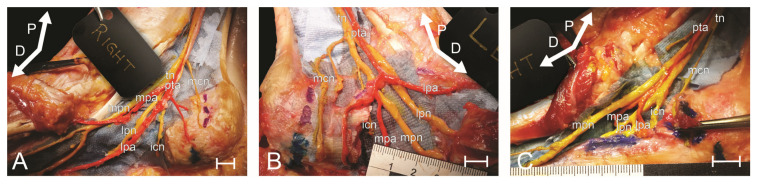
Medial view of three (**A**–**C**) different foot specimens demonstrating the neurovascular anatomy of the medial plantar and tarsal tunnel regions. The flexor retinaculum has been removed to provide an improved overview of the underlying structures. Orientation: P = proximal, D = distal. Abbreviations: tn = tibial nerve, pta = posterior tibial artery, mcn = medial calcaneal nerve, icn = inferior calcaneal nerve, lpn = lateral plantar nerve, mpn = medial plantar nerve, lpa = lateral plantar artery, mpa = medial plantar artery.

Since the flexor retinaculum is incised during S-PMR, the relative position of neurovascular branching points in relation to it was evaluated (see Figure 2A–C). A larger mean distance was observed between the origin of the medial calcaneal nerve relative to the superior border of the flexor retinaculum (51.2 mm). In contrast, bifurcation of the tibial nerve and the origin of the inferior calcaneal nerve relative to the superior border of the flexor retinaculum measured 16.4 mm and 6.0 mm, respectively. In many specimens, the inferior calcaneal nerve originated beneath or distal to the flexor retinaculum, resulting in negative measurement values. Additionally, the mean distance between the tibial nerve bifurcation and the origin of the inferior calcaneal nerve measured 10.45 mm. Analysis of potential sex-specific differences did not reveal any difference between division heights of the posterior tibial artery (*p* = 0.97) or the tibial nerve (*p* = 0.45). Similarly, no sex difference was observed in the origin level of the medial calcaneal nerve (*p* = 0.44). In contrast, the inferior calcaneal nerve did present a sex-related difference in origin level (*p* = 0.0020), indicating that its branching position might vary between male and female specimens. This finding should be interpreted cautiously given the exploratory study design and limited sample size.

## 4. Discussion

The anatomical origin of the abductor hallucis muscle has been described inconsistently across standard anatomical books. Most classical descriptions report two to three origins: the medial process of the calcaneal tuberosity and the plantar aponeurosis [20], with the third varying within the literature with attachments to structures such as the flexor retinaculum or the navicular bone [9,12,21]. This highlights the lack of consensus regarding the precise origin of this muscle. Despite its clinical relevance, particularly in surgical procedures involving the medial plantar region, the abductor hallucis origins have been relatively underexplored in the literature. For example, Wong et al. [22] conducted cadaveric dissections and reported a consistent origin in all specimens from the medial calcaneal tuberosity as well as from the deep fascia overlying the flexor digitorum brevis muscle. While this finding suggests a degree of anatomical uniformity, we, in contrast, found a greater degree of variability, identifying up to four origins of the abductor hallucis muscle. Notably, the first and fourth origins were consistently present across all specimens, whereas the first origin exhibited further sub-variants (Types Ia, Ib and Ic) underscoring the complexity of its morphology. Mizuno et al. [23] investigated the relationship between the abductor hallucis and the plantar aponeurosis, specifically analyzing the attachment or non-attachment to the central band of the aponeurosis, and reported associated sex-based differences. In contrast, the findings of the present study did not reveal any significant sex-related differences in the frequency or distribution of the identified origins. Taken together, these findings suggest that the abductor hallucis origins are more variable than traditionally described. This variability has important clinical implications in surgical approaches such as the S-PMR, where precise anatomical knowledge is essential to minimize the risk of iatrogenic injury.

The S-PMR is performed in a region of the plantar foot that lies in close proximity to multiple neurovascular structures. These include the medial and lateral plantar nerve and artery along with its branches and the medial and inferior calcaneal nerves. Iatrogenic injury to these structures can result in substantial functional morbidity. In light of these risks, the present study aimed to evaluate the spatial relationship between key neurovascular structures and the origins of the abductor hallucis, in addition to established anatomical reference points such as the flexor retinaculum and plantar aponeurosis.

Previous studies have already highlighted the limited safety margins in this region. For example, Hofmeister et al. [24] reported a distance of 15.5 mm between the plantar aponeurosis release site and the lateral plantar nerve during endoscopic fasciotomy procedures. Similarly, Yilmaz et al. [25] demonstrated that multiple neurovascular structures lie in close proximity to the surgical portal, with mean distances of 15.21 mm to the lateral plantar nerve and 10.46 mm to the inferior calcaneal nerve, underscoring the close relationship between the surgical field and adjacent neurovascular structures.

**Figure 3:**
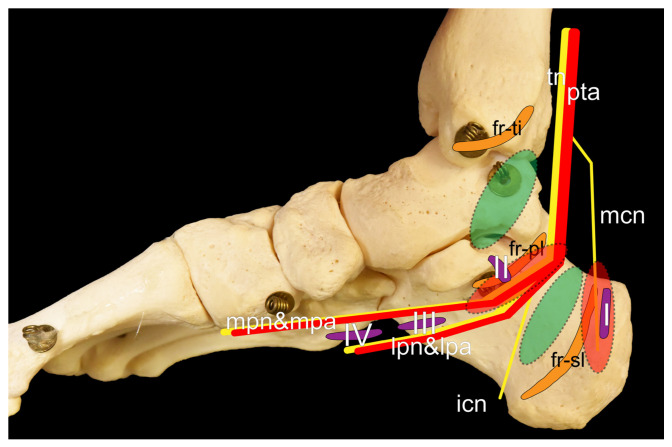
Medial view of an exemplary foot skeleton with an abstracted illustration of the neurovascular structures demonstrating relatively low- (highlighted in green) and high- (highlighted in red) risk zones for the S-PMR. The course of the tibial nerve and its branches, as well as the posterior tibial artery and its branches, is shown in relation to the abductor hallucis origins. Abbreviations: tn = tibial nerve, pta = posterior tibial artery, mcn = medial calcaneal nerve, icn = inferior calcaneal nerve, lpn = lateral plantar nerve, mpn = medial plantar nerve, lpa = lateral plantar artery, mpa = medial plantar artery, green ovals = low-risk zones, red ovals = high-risk zones, I–IV = origins of abductor hallucis I–IV, fr-ti = flexor retinaculum-tibial insertion, fr-pl = flexor retinaculum-profound layer, fr-sl = flexor retinaculum-superficial layer.

Consistent with prior literature, the present study demonstrates that multiple neurovascular structures lie in close proximity to the area of surgical release [24,26,27]. Building on these observations, distinct neurovascular “high-risk” zones can be defined based on their spatial relationships to the abductor hallucis origins (see Figure 3). The first “high-risk” zone is located at origin I of the abductor hallucis muscle, where the medial calcaneal nerve was found to be in close proximity. The median distance between origin I and the medial calcaneal nerve measured 3.2 mm. Notably, in several specimens, the nerve crossed directly over origin I or its sub-origins, causing a higher risk of injury during detachment. This identifies the region surrounding origin I as particularly vulnerable and necessitating caution during surgical release. A second “high-risk” zone was identified at origin II, corresponding to the region of bifurcation of the tibial nerve and the posterior tibial artery into their plantar branches. Here, multiple neurovascular structures were closely related to the muscle origin. The medial and lateral plantar nerves were located at close proximity to origin II, with measured distances of 3.0 mm and 5.3 mm, respectively, while the corresponding arteries were more distant at 7.3 mm and 6.8 mm. Importantly, the direct crossing of neurovascular structures over the muscle origins was observed in multiple specimens. This occurred three times between origin II and the medial plantar nerve and twice between origin II and the lateral plantar nerve, further increasing surgical risk in the area. Although origins 3 and 4 are in close proximity to surrounding neurovascular structures, they carry a lower risk of injury due to their greater distance from the surgical release site and are therefore not categorized as localized high-risk zones.

Based on these findings, potential relative “low-risk” zones for surgical access can be proposed. These include the medial aspect of the calcaneus, situated between origin I and the neurovascular bundle, as well as the region near the medial malleolus that lies medial to the entire neurovascular bundle. Awareness of these safer corridors may help reduce neurovascular injury during S-PMR. Knowledge of these anatomical “low-risk” zones may assist surgical planning by guiding incision placement and soft-tissue dissection away from regions of close neurovascular proximity.

This study has some limitations to consider. The sample size, while typical for anatomical research, was relatively limited. Furthermore, the analysis was based on elderly body donors—as a result, direct applicability to pediatric populations may be restricted. In addition, cadaveric anatomy may not fully reflect living tissue conditions or surgical positioning. The proposed “risk zones” were anatomically and morphologically defined based on cadaveric dissections and were not clinically validated. Moreover, paired specimens were included without adjustment for within-donor correlation. Due to the descriptive nature of the study design, the findings should be interpreted as exploratory observations rather than definitive evidence. The results nonetheless show the intimate topographical relationship between the origins of the abductor hallucis muscle and the neurovascular bundles relevant for all surgical approaches in this region.

## 5. Conclusions

In summary, this study reinforces the concept that the plantar surgical field is characterized by narrow safety margins and highlights anatomically defined relative “high-risk” zones that may require particular attention during operative planning and execution. Furthermore, the observed high variability and notable interindividual differences in the course and proximity of neurovascular structures emphasize the importance of a personalized, patient-specific approach. Such variability underscores the need for careful intraoperative assessment and individualized surgical planning to minimize the risk of iatrogenic injury.

## Figures and Tables

**Table 1:** Frequency of abductor hallucis origins and sub-origins. Values are presented as absolute numbers with corresponding percentages.

Structure	n	Frequency (%)
Origin I	41	100
Bifid sub-origin I	10	24
Trifid sub-origin I	2	5
Origin II	31	76
Origin III	28	68
Origin IV	41	100

**Table 2:** Distances between abductor hallucis origins and adjacent res. All distances are listed with full ranges. “n” indicates the number of measured specimens for the respective anatomical relationship.

Nerve	Presence of Nerve	Reference Origin	n	Distance (mm) *	Range (mm)	Nerve Crossing Over Origin
Medial plantar nerve	41/41	I	41	25.7	15.1–34.6	-
		II	31	3.0	0–18.7	3 specimens
		III	28	5.0 ± 5.2	4.0–37.2	-
		IV	41	5.1	0–47.5	-
Medial calcaneal nerve	40/41	I	40	3.2	0.2–14.2	-
		II	30	16.5 ± 10.5	0.5–36.3	-
		III	27	34.0	17.0–51.7	-
		IV	40	36.1	1.6–59.7	-
Lateral plantar nerve	41/41	I	41	17.3	0.2–26.5	-
		II	31	5.3 ± 5.4	0–19.0	2 specimens
		III	28	14.1 ± 6.1	0–28.3	-
		IV	41	2.6 ± 2.9	0–10.2	-
Inferior calcaneal nerve	39/41	I	39	14.0 ± 3.9	6.1–20.4	-
		II	30	7.0 ± 6.1	0–23.0	1 specimen
		III	27	20.0 ± 5.4	11.7–34.1	-
		IV	39	19.3 ± 12.6	0–52.8	-

* Normally distributed variables are presented as mean ± SD; non-normally distributed variables are presented as median values. Measurements were only performed when the respective origin and neurovascular structure were identifiable. Only directly observed overlaps were recorded.

**Table 3:** Distances between abductor hallucis origins and adjacent arteries. All distances are listed with full ranges. “n” indicates the number of measured specimens for the respective anatomical relationship.

Artery	Presence of Artery	Reference Origin	n	Distance (mm) *	Range (mm)	Artery Crossing Over Origin
Medial plantar artery	41/41	I	41	24.6 ± 6.1	13.2–37.1	-
		II	31	7.3	0–27.4	-
		III	28	9.1	0–28.4	-
		IV	41	6.9	0–24.1	-
Lateral plantar artery	41/41	I	41	17.7 ± 7.2	0–32.0	-
		II	31	6.8	0–16.7	-
		III	28	14.6 ± 5.2	5.9–32.9	-
		IV	41	5.0 ± 5.1	0–17.6	-

* Normally distributed variables are presented as mean ± SD; non-normally distributed variables are presented as median values. Measurements were only performed when the respective origin and neurovascular structure were identifiable. Only directly observed overlaps were recorded.

## Data Availability

The original contributions presented in this study are included in the article. Further inquiries can be directed to the corresponding author.

## Abbreviations

The following abbreviations are used in this manuscript:

fr-pl	flexor retinaculum-profound layer
fr-sl	flexor retinaculum-superficial layer
fr-ti	flexor retinaculum-tibial insertion
icn	inferior calcaneal nerve
lpa	lateral plantar artery
lpn	lateral plantar nerve
mcn	medial calcaneal nerve
mpa	medial plantar artery
mpn	medial plantar nerve
pa	plantar aponeurosis
pta	posterior tibial artery
SD	standard deviation
S-PMR	superficial plantar-medial release
tn	tibial nerve

## References

[1] Feng S.-M., Song R.-L., Wang A.-G., Sun Q.-Q., Zhang S.-C. (2021). Endoscopic Partial Plantar Fasciotomy via 2 Medial Portals vs Mini-Open Procedure for Refractory Plantar Fasciitis. Foot Ankle Int..

[2] Yanbin X., Haikun C., Xiaofeng J., Wanshan Y., Shuangping L. (2015). Treatment of Chronic Plantar Fasciitis with Percutaneous Latticed Plantar Fasciotomy. J. Foot Ankle Surg..

[3] Buchanan B.K., Sina R.E., Kushner D. (2026). ‘Plantar Fasciitis’. StatPearls.

[4] Ohuchi H., Ichikawa K., Shinga K., Hattori S., Yamada S., Takahashi K. (2013). Ultrasound-assisted endoscopic partial plantar fascia release. Arthrosc. Tech..

[5] Abarca M., Filippi J., Wagner Hitschfeld E., Wagner Hitschfeld P. (2022). Plantar Fasciitis. Foot and Ankle Disorders.

[6] Younger A.S.E., Hansen S.T. (2005). Adult Cavovarus Foot. J. Am. Acad. Orthop. Surg..

[7] Mosca V.S. (2001). The cavus foot. J. Pediatr. Orthop..

[8] Mousafeiris V., Dreyer M.A., Thomas A. (2026). ‘Pediatric Foot Alignment Deformities’. StatPearls.

[9] Mosca V.S. (2014). Principles and Management of Pediatric Foot and Ankle Deformities and Malformations.

[10] Padgett A.M., Kothari E., Conklin M.J. (2024). Two-stage corrective operation for the treatment of pes cavovarus in patients with spina bifida. World J. Orthop..

[11] Myerson M.S., Li S., Wagner Hitschfeld E., Wagner Hitschfeld P. (2022). Cavus Foot. Foot and Ankle Disorders.

[12] Streicher J., Pretterklieber M.L., Waldeyer A., Anderhuber F., Pera F., Streicher J. (2012). 4 Bewegungsapparat. 4.4 Untere Extremität, Membrum inferius. Waldeyer-Anatomie des Menschen.

[13] Boyle R.A., Slater G.L. (2003). Endoscopic Plantar Fascia Release: A Case Series. Foot Ankle Int..

[14] Malahias M.-A., Cantiller E.B., Kadu V.V., Müller S. (2020). The clinical outcome of endoscopic plantar fascia release: A current concept review. Foot Ankle Surg..

[15] Liew S.K., Saw A., Chua Y.P. (2022). Foot Arch Changes after Endoscopic Plantar Fascia Release for Recalcitrant Plantar Fasciitis. Malays. Orthop. J..

[16] Oh S.J., Kwon K.H., Hah J.S., Kim D.E., Demirci M. (1999). Lateral plantar neuropathy. Muscle Nerve.

[17] Diers D.J. (2008). Medial calcaneal nerve entrapment as a cause for chronic heel pain. Physiother. Theory Pract..

[18] Lew J.T., Stearns M. (2026). ‘Tibial Neuropathy’. StatPearls.

[19] Mazzotti A., Zielli S.O., Artioli E., Facchini G., Miceli M., Faldini C. (2022). Iatrogenic Lesion of the Lateral Plantar Artery following Plantar Fasciotomy for Cavus Foot Correction—A Case Report. J. Orthop. Case Rep..

[20] Schünke M., Schulte E., Schumacher U., Voll M., Wesker K.H. (2022). Prometheus Allgemeine Anatomie und Bewegungssystem: LernAtlas der Anatomie.

[21] Aumüller G. (2007). Anatomie. Duale Reihe.

[22] Wong Y.S. (2007). Influence of the Abductor Hallucis Muscle on the Medial Arch of the Foot: A Kinematic and Anatomical Cadaver Study. Foot Ankle Int..

[23] Mizuno D., Otsuka S., Shan X., Umemoto K., Naito M. (2024). Variation in the origin of the plantar aponeurosis and its relationship to the origin of the abductor hallucis muscle. Clin. Anat..

[24] Hofmeister E.P., Elliott M.J., Juliano P.J. (1995). Endoscopic Plantar Fascia Release: An Anatomical Study. Foot Ankle Int..

[25] Yilmaz E., Yozgatli T.K., Aktekin A., Ozturk O., Aktekin M., Kocaoglu B. (2025). Safety and Feasibility of the Plantar Portal Technique in the Surgical Management of Plantar Fasciitis: A Cadaveric Study. J. Foot Ankle Res..

[26] Kiskaddon E.M., Meeks B.D., Roberts J.G., Laughlin R.T. (2018). Plantar Fascia Release Through a Single Lateral Incision in the Operative Management of a Cavovarus Foot: A Cadaver Model Analysis of the Operative Technique. J. Foot Ankle Surg..

[27] Çatal B., Keskinbora M., Keskinöz E.N., Tümentemur G., Azboy İ., Demiralp B. (2019). Percutaneous Plantar Fascia Release with Needle: Anatomic Evaluation with Cadaveric Specimens. J. Foot Ankle Surg..

